# Myocardial remodeling in valvular heart disease: pathophysiology, multimodality imaging and treatment implications

**DOI:** 10.1007/s10741-026-10660-0

**Published:** 2026-07-30

**Authors:** Maximilian Autherith, Laurenz Hauptmann, Raghuram Palaparti, Philipp E. Bartko, Christian Nitsche

**Affiliations:** Department of Internal Medicine II, Division of Cardiology, Medical University of Vienna, Waehringerguertel 18-20, Vienna, 1090 Austria

## Abstract

Valvular cardiomyopathy has emerged as a key concept linking valvular heart disease (VHD) with myocardial remodeling and the development of heart failure (HF). This state-of-the-art review provides a comprehensive overview of pathophysiological mechanisms, multimodality imaging, and implication for valve interventions across the spectrum of VHD. We systematically address HF in the context of aortic valve disease, including aortic stenosis and aortic regurgitation, mitral valve disease with a particular focus on mitral regurgitation, and tricuspid valve regurgitation. For each valvular lesion, we integrate evidence on pathophysiological mechanisms on myocardial (mal)adaptation leading to myocardial fibrosis and ultimately HF and death. Additionally, we highlight the role of multimodality imaging for early detection of myocardial remodeling and discuss implications for patient selection and timing of valve interventions. Beyond single-valve disease, we further explore the complex interplay of multiple and mixed valvular heart disease and review emerging data on the interaction between VHD and cardiac amyloidosis, an increasingly recognized and clinically relevant overlap. Overall, this state-of-the-art review underscores that outcomes in VHD are not solely determined by severity of valvular lesions but critically depend on the extent of associated myocardial damage. A deeper understanding of valvular cardiomyopathy may improve risk stratification, refine patient selection, and support the implementation of tailored therapeutic strategies.

## Introduction

Valvular heart disease (VHD) represents a major cause of cardiovascular morbidity and mortality worldwide. Traditionally, clinical decision-making in VHD has focused primarily on the severity of valvular lesions and respective hemodynamic consequences. Therefore, current guideline recommendations for the management of VHD largely rely on parameters such as severity of VHD, ventricular size, and systolic function [1, 2].

However, increasing evidence suggests that the myocardium plays a pivotal role in determining symptoms, functional capacity, and long-term outcomes in patients with VHD. Chronic pressure or volume overload imposed by valvular lesions leads to a complex process of myocardial remodeling involving left ventricular hypertrophy and/or dilatation, interstitial and/or focal myocardial fibrosis, and progressive ventricular dysfunction. Beyond the left ventricle, the remodeling process in VHD may involve atrial, pulmonary vascular, and right ventricular (RV) adaptations. While the myocardial consequences and stages of cardiac damage in severe aortic stenosis (AS) as well as the consequences of pressure overload relief through aortic valve replacement (AVR) have been extensively studied [3–12] similar remodeling processes may also be seen in mitral and tricuspid valve disease, but are less well described. Moreover, patients with mixed or multiple valvular heart disease (MMVD) may develop particularly complex patterns of myocardial adaptation and damage due to the combined hemodynamic burden. Acknowledgement of these maladaptive alterations has led to the emerging concept of valvular cardiomyopathy, in which myocardial disease develops secondary to VHD.

In this context, valvular cardiomyopathy should be understood as a disease continuum linking chronic valvular loading conditions with myocardial, atrial, pulmonary vascular, RV, and systemic consequences. Depending on the type, severity, and duration of the valvular lesion, patients may develop heart failure (HF) phenotypes characterized predominantly by diastolic dysfunction, myocardial stiffening, atrial remodeling, and congestion or, alternatively, by progressive ventricular dilatation and systolic dysfunction. Importantly, valvular cardiomyopathy may persist even after successful valve intervention, as irreversible myocardial fibrosis, atrial cardiomyopathy, pulmonary vascular remodeling, RV dysfunction, and end-organ injury may limit reverse remodeling and contribute to residual HF symptoms and adverse outcomes. Thus, the concept extends beyond ventricular remodeling alone and provides an integrative framework to understand the transition from isolated VHD to multi-organ HF syndromes [13, 14].

**Fig. 1:**
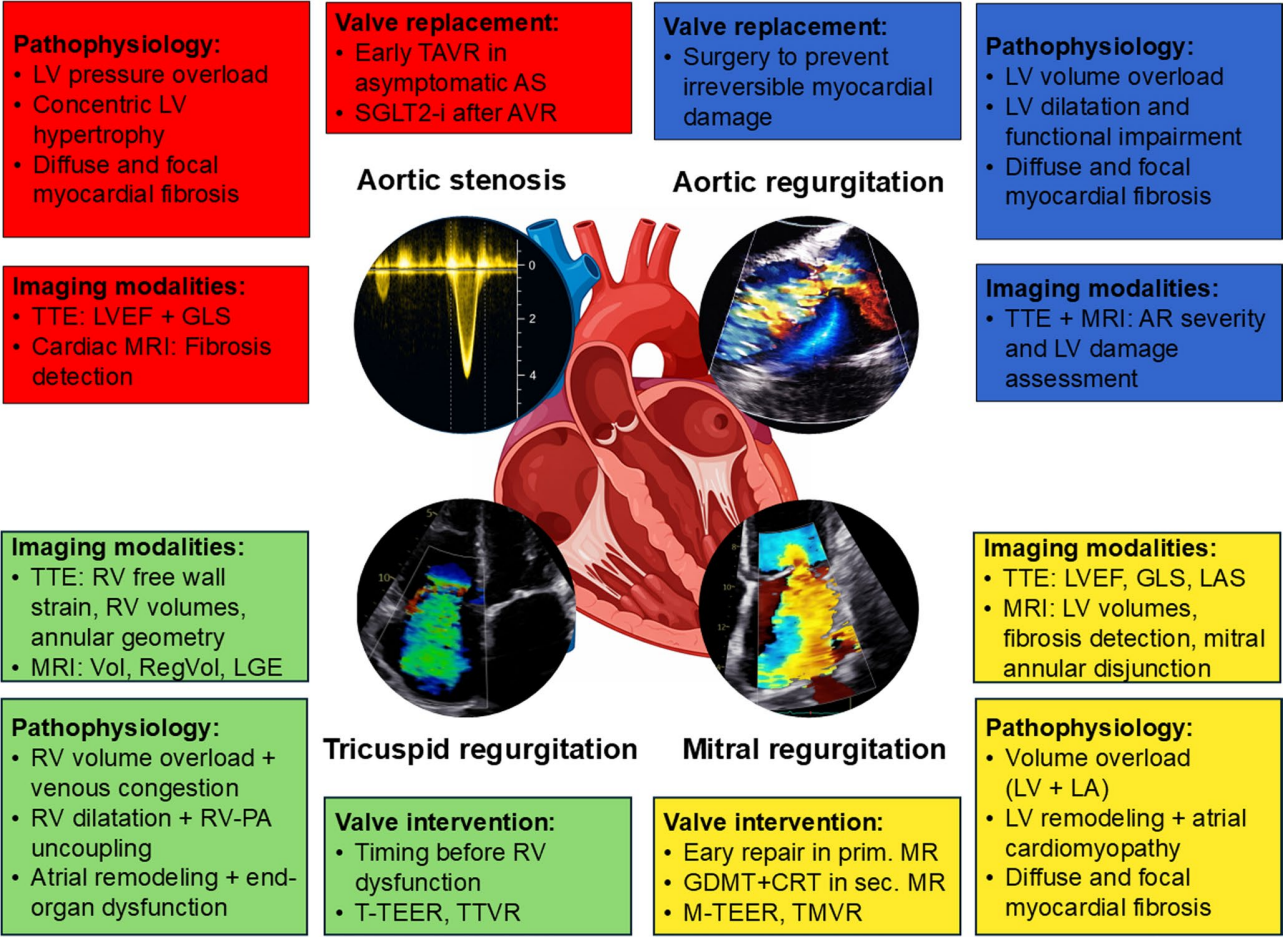
Central Illustration: Myocardial Remodeling in Valvular Heart Disease: Pathophysiology, multimodality imaging and treatment implications This state-of-the-art review summarizes the current understanding of pathophysiological mechanisms of myocardial remodeling, highlights the role of multimodality imaging for myocardial characterization, and addresses the clinical implications of myocardial injury across the spectrum of valvular heart disease

In this state-of-the-art review, we summarize the current understanding of valvular cardiomyopathy across the spectrum of VHD. We discuss the pathophysiological mechanisms of myocardial remodeling in aortic, mitral, and tricuspid valve disease, highlight the role of multimodality imaging for myocardial characterization, and address the clinical implications of myocardial injury in patients with single and multiple valvular lesions. The hallmarks of valvular cardiomyopathy across the spectrum of VHD are summarized in Table 1 and the Central Illustration (Fig. 1).

**Table 1:** Hallmarks of valvular cardiomyopathy across the spectrum of valvular heart disease

Valve lesion	Loading condition	Cardiomyopathy phenotype	Key markers of remodeling	Markers of advanced cardiomyopathy	Therapeutic implication
Aortic stenosis	Chronic LV pressure overload	Predominant concentric LV remodeling, diffuse and focal fibrosis	LV mass, GLS, diastolic dysfunction (E/e′, LAVI, LA strain), CMR-ECV/LGE	Focal fibrosis, impaired GLS, advanced cardiac damage	Early AVR before irreversible myocardial damage develops
Aortic regurgitation	Chronic LV volume overload	Eccentric LV remodeling with progressive fibrosis	LV volumes, GLS, CMR regurgitant fraction and myocardial fibrosis	LV dilatation, declining LVEF, myocardial fibrosis, advanced cardiac damage	AVR before irreversible LV remodeling and systolic dysfunction
Primary mitral regurgitation	Chronic LV/LA volume overload	Eccentric LV remodeling and atrial cardiomyopathy	LV volumes, GLS, LA strain, CMR fibrosis	Reduced contractile reserve, pulmonary hypertension, myocardial fibrosis	Early mitral repair before irreversible LV dysfunction
Ventricular functional mitral regurgitation	LV remodeling due to cardiomyopathy	LV cardiomyopathy with leaflet tethering	LV volumes, GLS, tethering geometry, myocardial scar/fibrosis	LV (and RV) dysfunction, pulmonary hypertension	Optimize GDMT/CRT, TEER in selected patients
Atrial functional mitral regurgitation	Chronic LA pressure/volume overload	Atrial cardiomyopathy	LA size, LA strain, annular dilatation	Atrial remodeling, pulmonary hypertension	Rhythm control, HFpEF therapy, TEER in selected patients
Mitral stenosis	Chronic LA pressure overload	Atrial, pulmonary vascular, and RV cardiomyopathy	MV area, LA size, PAP, RV function	Pulmonary vascular remodeling, RV dysfunction	Commissurotomy or surgery before irreversible pulmonary vascular disease
Tricuspid regurgitation	Chronic RA/RV volume overload	Right-sided cardiomyopathy	RV free-wall strain, RV-PA coupling, RA remodeling, venous congestion, CMR RV volumes	Severe RV dysfunction, RV-PA uncoupling, hepatic/renal dysfunction	Early intervention before advanced right-heart failure
Multiple and mixed VHD	Combined pressure and volume overload	Multi-chamber cardiomyopathy	Multimodality imaging (TTE, TEE, CMR, CT, invasive hemodynamics)	Multi-chamber remodeling, pulmonary hypertension, end-organ dysfunction	Identify dominant lesion, individualized Heart Team strategy

*AVR* indicates aortic valve replacement, *CMR* cardiac magnetic resonance, *CRT* cardiac resynchronization therapy, *CT* computed tomography, *ECV* extracellular volume fraction, *E/e′* ratio of early mitral inflow velocity to early diastolic mitral annular velocity, *GDMT* guideline-directed medical therapy, *GLS* global longitudinal strain, *HFpEF* heart failure with preserved ejection fraction, *LA* left atrium, *LAVI* left atrial volume index, *LGE* late gadolinium enhancement, *LV* left ventricle,* LVEF* left ventricular ejection fraction, *MV* mitral valve, *PAP* pulmonary artery pressure, *RA* right atrium, *RV* right ventricle, *RV-PA* right ventricular-pulmonary artery, *TEER* transcatheter edge-to-edge repair, *TEE* transoesophageal echocardiography, *TTE* transthoracic echocardiography, *VHD* valvular heart disease

## Aortic valve disease

### Aortic stenosis

#### Pathophysiology and myocardial remodeling

AS represents the most common VHD in the elderly population and is characterized by progressive obstruction to left ventricular outflow at the level of the aortic valve due to calcific degeneration. Beyond the mechanical obstruction, AS imposes a pressure overload on the left ventricle (LV) that induces a complex process of myocardial remodeling. Increasing evidence suggests that myocardial remodeling, induced by AS plays a pivotal role in the clinical course of the disease [15]. The hallmark of AS-related myocardial remodeling is concentric left ventricular hypertrophy, which initially develops as an adaptive response to normalize wall stress in the presence of increased afterload. Sustained pressure overload ultimately triggers a maladaptive cascade that profoundly affects cardiac structure and function including cardiomyocyte hypertrophy, interstitial fibrosis, microvascular dysfunction, and alterations in myocardial energetics [15, 16]. Myocardial fibrosis has emerged as a pathophysiological hallmark of AS-induced cardiomyopathy. Diffuse interstitial fibrosis develops early in the disease process and reflects increased collagen deposition within the extracellular matrix. As myocardial injury progresses, focal fibrosis occurs, which represents irreversible myocardial damage and contributes to ventricular decompensation and death [5–7, 17, 18].

#### Multimodality imaging to assess myocardial damage

Multimodality imaging techniques play a crucial role in the assessment of myocardial remodeling and damage in patients with AS. Transthoracic echocardiography (TTE) remains the primary imaging modality for the diagnosis and grading of AS severity [1]. Beyond left ventricular ejection fraction (LVEF), global longitudinal strain (GLS) and markers denoting diastolic dysfunction such as E/e’, left atrial volume index and left atrial strain may be altered in the context of increased myocardial stiffening and have been demonstrated to convey prognostic information in AS. Moreover, GLS has the potential to detect systolic dysfunction prior to a decline of LVEF and therefore represents a more sensitive marker of impaired contractility [19–21].

Additionally, cardiac magnetic resonance imaging (CMR) has emerged as a powerful tool for the assessment of cardiac damage as the application of contrast and mapping techniques allow for in-depth tissue characterization otherwise inaccessible for echocardiography. Focal fibrosis is visualized by late gadolinium enhancement (LGE) and has been extensively described as a strong predictor of mortality in patients with AS. Quantitative mapping techniques allow for assessment of diffuse interstitial alterations not captured by LGE. Diffuse fibrosis has been demonstrated to follow an endo- to epicardial gradient, and thereby mirrors the pattern of myocardial hypoperfusion seen on stress CMR [18]. These observations have stimulated a randomized controlled trial that investigated a strategy of early intervention in patients with focal fibrosis seen on pre-interventional CMR, as discussed below [6–10, 18].

#### Implication on timing of valve intervention

The only effective treatment option for severe AS is aortic valve replacement (AVR), either performed surgically (SAVR) or through a transcatheter approach (TAVR). Current guideline recommendations for intervention are largely based on symptom status, AS severity, and the presence of left ventricular systolic dysfunction [1]. Historically, SAVR was the mainstay of treatment for severe symptomatic AS. However, more recently TAVR has emerged as the preferred treatment option for patients ≥ 70 years irrespective of surgical risk. In the context of valvular cardiomyopathy, accumulating evidence suggests that myocardial damage may develop before the conventional thresholds for aortic valve intervention are reached. Recently, these observations have stimulated growing interest in earlier intervention strategies in patients with asymptomatic severe AS or moderate AS. In the EVOLVED trial, patients with severe AS and no valve-related symptoms underwent CMR to detect focal myocardial fibrosis. If present, they were randomized to either active surveillance or an early intervention strategy. The trial did not meet its primary composite endpoint of all-caused death and/or unplanned AS-related hospitalization, but early intervention demonstrated a significant reduction in the time to first unplanned AS-related hospitalization. The EARLY TAVR trial investigated patients with asymptomatic severe AS and preserved LVEF who were randomized to active surveillance vs. early intervention [22]. The trial met its primary composite endpoint of death, stroke, or unplanned hospitalization for cardiovascular causes which was superior for the early intervention strategy. However, this result was mainly driven by the high conversion rate to intervention in the conservative arm within the first six months of randomization, which was considered to be an unplanned cardiovascular hospitalization [22]. The TAVR UNLOAD trial investigated patients with moderate AS and reduced LVEF. TAVR was not superior to active surveillance for the composite hierarchical endpoint of all-cause death, stroke, disease-related hospitalizations, and change from baseline in quality of life scores [23]. These results need to be interpreted with caution as the trial had difficulties with recruitment and therefore was rather small (~ 90 patients per arm). Currently, the large PROGRESS (NCT04889872) and the EXPAND TAVR II (NCT05149755) trials are ongoing and investigate the effectiveness of TAVR in patients with moderate AS and preserved LVEF. Findings from these trials will improve our understanding of the ongoing search for the optimal timing of aortic valve intervention in AS.

#### Heart failure management in AS

From our current understanding HF management in the presence of AS does not differ from other HF entities and international HF guidelines apply. However, it may be hypothesized that structural changes associated with reverse remodeling in AS give rise to a phenotype that differs significantly in terms of pathogenesis, myocardial substrate, and possibly also pharmacological treatability, from other HF subtypes. This also raises the question of optimal supportive HF management after successful valve replacement. Clearly, a residual HF component remains following afterload removal in a considerable proportion of patients as indicated by symptoms, biomarkers and structural alterations despite reverse myocardial remodeling [13]. Recently, randomized controlled trials investigated the efficacy of HF treatment strategies in patients treated with AVR for severe AS. Dapagliflozin demonstrated favorable effects in severe AS patients with a history of HF for the composite of all-cause death and hospitalization for HF at 1-year after TAVR. This effect was driven by HF events as no significant differences in all-cause mortality were observed [24]. The RAS TAVI trial investigated the effectiveness of ramipril in reducing the composite of death, HF hospitalization and stroke at 1-year after TAVR. The trial was negative, but again, significant reductions in the rate of HF readmissions were observed for the ramipril group [25]. Finally, the EASE-TAVR trial investigated an individualized decongestive treatment approach compared to decongestion as per clinical judgement alone in hypervolemic patients with severe AS undergoing TAVR. The trial demonstrated significant favorable effects of individualized decongestive treatment for the composite of all-cause mortality and hospitalization for HF at 12 months and indicated a tendency toward reduced all-cause mortality with prolonged follow-up until 36 months [26, 27].

### Aortic regurgitation

#### Pathophysiology and myocardial remodeling

Aortic regurgitation (AR) imposes a hemodynamic burden on the LV characterized primarily by volume overload. Regurgitant blood flow from the aorta into the LV leads to increased end-diastolic volume and elevated stroke volume requirements to maintain cardiac output. This persistent volume overload initiates a complex process of myocardial remodeling that ultimately determines clinical outcomes in patients with AR.^28–30^ In order to meet the demand of higher stroke volume created by an increase in preload, the LV undergoes dilatation and eccentric hypertrophy. This adaptive remodeling pattern allows patients to remain asymptomatic for many years despite substantial structural alterations [28]. However, sustained hemodynamic stress eventually drives the transition from compensated to maladaptive remodeling leading to increased wall stress, myocardial oxygen demand, and myocardial fibrosis [28]. Diffuse and focal interstitial fibrosis contribute to impaired ventricular compliance and declining contractile reserve. This process marks the transition to ventricular decompensation and is associated with declining systolic function, the onset of symptoms and ultimately death [31].

#### Multimodality imaging to assess myocardial damage

Accurate quantitative assessment of AR represents a considerable challenge in daily clinical practice. In this scenario, multimodality imaging plays a pivotal role for diagnosis, risk stratification, and treatment planning. TTE remains the first-line modality for diagnosis, allowing evaluation of regurgitation severity, ventricular size, systolic function, and hemodynamic consequences. Key parameters of (semi-)quantitative assessment include regurgitant volume, regurgitant fraction, vena contracta width, and pressure half time, remodeling parameters include left ventricular dimensions, and LVEF is mainly used as a functional parameter, as emphasized by current guideline recommendations [1]. However, LVEF often remains normal or even supranormal during the compensated phase of the disease, limiting its sensitivity for detecting early myocardial dysfunction. Advanced echocardiographic techniques such as GLS can detect subtle impairment of myocardial function before conventional measures deteriorate and may provide incremental prognostic information [32].

Echocardiographic evaluation of AR requires complex multi-parametric assessment and is often complicated by eccentric regurgitant jet anatomy. CMR has emerged as a particularly valuable tool to objectify AR severity and is less prone to inter-observer variability. CMR provides highly reproducible quantification of ventricular volumes and regurgitant volume/flow. Furthermore, it allows for myocardial tissue characterization which may expose adverse features of myocardial remodeling including areas of diffuse and focal myocardial fibrosis. Recent investigations have further highlighted the prognostic value of CMR-based remodeling. Importantly, LV volume parameters outperformed LV dimension parameters and mitral valve/left atrial, and right heart identified patients with excessive mortality risk [33, 34].

#### Implication on timing of valve intervention

The primary treatment for severe AR is SAVR or, in selected cases, valve repair. Transcatheter techniques are increasingly used for patients at high or prohibitive surgical risk. Current guideline recommendations emphasize symptom status and the presence of ventricular dysfunction or ventricular dilation exceeding guideline thresholds as the principal triggers for intervention. In symptomatic patients with severe AR, surgery is recommended irrespective of left ventricular function. In asymptomatic individuals, intervention is generally advised once LVEF declines or left ventricular end-systolic dimensions exceed guideline thresholds, reflecting the onset of ventricular decompensation [1].

These thresholds aim to identify the optimal window for intervention before irreversible myocardial damage develops. Imaging-biomarkers not mentioned in current guidelines, such as LV myocardial strain and myocardial fibrosis markers hold promise to identify patients at increased mortality risk despite fulfilling no guideline indications for interventions [29]. Whether these markers will be implemented to guide a strategy of early valve interventions remains to be investigated [29, 32].

Following successful valve replacement, significant reverse ventricular remodeling is often observed. Reduction of volume overload leads to decreases in ventricular size and (partial) regression of hypertrophy. The extent of recovery is strongly influenced by the degree of pre-interventional myocardial damage. Patients with advanced fibrosis or long-standing ventricular dilatation may demonstrate incomplete recovery of systolic function and remain at increased risk for HF and death despite successful AVR [30, 33].

#### Heart failure management in AR

Similar to AS, conventional HF medication bears the potential to aid the reverse remodeling process in AR patients after AVR. However, due to the lack of data, no specific recommendations currently exist. Unless proven otherwise, HF management in this scenario should follow current HF guidelines [35].

## Mitral valve disease

### Mitral regurgitation

#### Pathophysiology and myocardial remodeling

Mitral regurgitation (MR) is a heterogeneous substrate of volume overload-driven valvular cardiomyopathy. Three distinct entities should be recognized: primary MR, ventricular functional MR and atrial functional MR, each characterized by distinct pathophysiological mechanisms, remodeling patterns, and therapeutic implications. Primary MR results from structural abnormalities of the leaflets, chordae, papillary muscles, or annulus, most commonly due to degenerative disease in high-income countries and due to rheumatic disease globally [36]. In contrast, secondary MR reflects failure of structurally normal leaflets to coapt because of myocardial remodeling and can be further subdivided into ventricular functional MR and atrial functional MR [37]. Ventricular functional MR accompanies ischemic or nonischemic cardiomyopathy, whereas atrial functional MR arises from annular dilation and left atrial myopathy, typically in long-standing atrial fibrillation or HF with preserved ejection fraction [38–40].

MR induces cardiomyopathy primarily through chronic volume overload of the LV and left atrium. In the compensated phase, eccentric remodeling and increased chamber compliance preserve forward output despite regurgitant flow [41]. Persistent hemodynamic stress, however, promotes extracellular matrix remodeling, myocardial fibrosis, and loss of contractile reserve [42]. The LVEF may appear preserved despite (sub-)clinical myocardial dysfunction as the regurgitant orifice lowers effective afterload. MR also drives progressive atrial remodeling including left atrial dilation and the onset of atrial fibrillation, which in turn aggravate annular dilation and worsen regurgitation. Atrial remodeling in this context reflects the development of atrial cardiomyopathy, characterized by structural, functional, and electrophysiological abnormalities of the atrial myocardium [43]. In atrial functional MR, atrial cardiomyopathy associated with long-standing atrial fibrillation or HF with preserved ejection fraction leads to annular dilation and leaflet malcoaptation despite preserved ventricular function [44]. Rising left atrial pressure is transmitted to the pulmonary circulation, leading to pulmonary vascular remodeling, pulmonary hypertension, and eventually RV dysfunction [45]. Thus, MR is not merely a valvular lesion but a multi-chamber cardiomyopathy affecting the entire cardiopulmonary unit [46].

#### Multimodality imaging to assess myocardial damage

In MR, early identification of myocardial involvement is essential to prevent irreversible remodeling. TTE remains the cornerstone of assessment, providing information on ventricular size and systolic function, atrial dimensions, pulmonary pressures, RV function, and lesion severity [47, 48]. However, ejection fraction alone is an insensitive marker of myocardial health in severe MR. GLS can detect (sub-)clinical contractile dysfunction before ejection fraction declines, while left atrial size, reservoir function, and strain provide complementary evidence of atrial cardiomyopathy and chronic hemodynamic burden [49].

CMR represents the gold standard for quantification of ventricular volumes and may identify focal or interstitial fibrosis by LGE or parametric mapping [50]. It is also useful for identifying mitral annular disjunction and detecting inferolateral myocardial fibrosis, associated with arrhythmic mitral valve prolapse. Current decision-making nevertheless remains anchored to guideline thresholds. In severe primary MR, intervention is recommended in symptomatic patients when a durable procedure is feasible. In asymptomatic severe primary MR, intervention is indicated once early left ventricular dysfunction appears [1, 51].

#### Implication on timing of valve intervention

Performing intervention for MR aims to reduce myocardial stress, prevent progression of ventricular and atrial remodeling, and improve outcomes through timely lesion correction. In primary MR, surgical mitral repair is preferred, because it preserves the sub-valvular apparatus, and is associated with better survival and reverse remodeling compared with conservative management. In high- or prohibitive-risk patients, mitral transcatheter edge-to-edge repair (M-TEER) provides an effective alternative for symptom relief and regurgitation reduction. In ventricular functional MR, treatment begins with guideline-directed medical HF therapy. Cardiac resynchronization therapy is important in eligible patients with dyssynchrony as restoration of coordinated ventricular contraction can reduce leaflet tethering and regurgitation. Major HF trials such as CARE-HF [52] , COMPANION [53], and MADIT-CRT [54] demonstrated reverse remodeling with secondary reductions in functional MR. In atrial functional MR, treatment focuses primarily on management of the underlying atrial cardiomyopathy, including rhythm or rate control in patients with atrial fibrillation [1, 2] and optimization of medical therapy for heart failure with preserved ejection fraction where appropriate. In selected patients with persistent severe atrial functional MR despite optimized medical and rhythm-control therapy mitral valve surgery or M-TEER may represent effective therapeutic options.

Early randomized evidence for M-TEER was provided by the EVEREST II trial [55], which included a predominant degenerative MR population (73%) and demonstrated that percutaneous mitral repair was safer than surgery but less effective in achieving durable MR reduction with comparable improvement in symptoms and no difference in long-term survival. Subsequently, the role of transcatheter repair in ventricular functional MR was evaluated in two randomized trials with diverging results. In the MITRA-FR trial [56], transcatheter repair did not improve mortality or HF hospitalizations, whereas the COAPT trial [57] demonstrated beneficial outcomes in respective endpoints. In the MITRA-FR trial, patients had relatively less severe MR but more advanced ventricular dilatation, suggesting that MR was proportionate to the degree of LV remodeling. In contrast, the COAPT trial enrolled patients with more severe MR and less advanced ventricular dilatation, consistent with disproportionate MR. These differences in baseline disease severity and ventricular geometry are considered key drivers of the divergent trial results and highlight that the benefit of transcatheter repair depends on the interplay between regurgitation severity and the underlying myocardial substrate [37, 58]. More recently, the RESHAPE-HF2 trial further expanded the evidence supporting M-TEER in patients with ventricular functional MR [59]. In contrast to COAPT, the trial enrolled a broader patient population with less restrictive inclusion criteria and demonstrated a reduction in cardiovascular death and HF hospitalization. These findings suggest that the benefit of transcatheter intervention may extend beyond the highly selected population enrolled in COAPT and challenge a strict dichotomous interpretation of the proportionate/disproportionate MR concept. Rather than relying on a single framework, contemporary patient selection increasingly integrates MR severity, ventricular size and function, optimization of guideline-directed medical therapy, pulmonary pressures, RV function, and overall clinical status. Patients with severe symptomatic MR despite optimized medical therapy, persistent HF symptoms, and preserved myocardial reserve appear most likely to benefit from intervention. Newer technologies, including transcatheter mitral valve replacement may further expand options in complex anatomy or advanced cardiomyopathy [1].

### Mitral stenosis

#### Pathophysiology and myocardial remodeling

Mitral stenosis (MS) is a distinct form of valvular cardiomyopathy in which cardiac remodeling is driven less by direct left ventricular pressure or volume overload and more by chronic left atrial pressure overload, pulmonary vascular remodeling, and progressive right-sided dysfunction. Rheumatic heart disease remains the dominant cause worldwide [60]. In higher-income countries, degenerative calcific MS due to severe mitral annular calcification is more common than rheumatic etiology and increasingly encountered [61].

The primary hemodynamic disturbance is obstruction to left ventricular inflow. Chronic elevation of left atrial pressure leads to atrial cardiomyopathy characterized by left atrial dilation, fibrosis, and progressive mechanical dysfunction which predisposes to the onset of atrial fibrillation and thromboembolism. Persistent pressure overload subsequently induces pulmonary vascular remodeling with a progressive increase in pulmonary vascular resistance, resulting in pulmonary vascular cardiomyopathy and progressive RV pressure overload. Over time, this promotes RV remodeling, dysfunction and ultimately right-sided HF. Left ventricular dysfunction is less prominent, but several mechanisms have been proposed, including chronic underfilling, myocardial involvement in rheumatic heart disease and coexistent coronary artery disease. Thus, the clinical course of MS is predominantly determined by the progressive interaction between atrial, pulmonary vascular, and RV cardiomyopathy rather than by the severity of mitral valve obstruction alone [62].

#### Multimodality imaging to assess myocardial damage

For the diagnosis, echocardiography remains the cornerstone for comprehensive evaluation of MS, enabling assessment of valve morphology, stenosis severity, and hemodynamic consequences [63]. In rheumatic MS, mitral valve area assessed by planimetry is the preferred method to define severity, with a threshold of ≤ 1.5 cm² indicating clinically significant disease when integrated with symptoms and markers of hemodynamic decompensation. Mean transvalvular gradient and pulmonary artery pressure reflect the downstream consequences, whereas assessment of RV function provides additional prognostic information in advanced disease [64]. In addition, left atrial dilatation represents a key marker of chronic pressure overload and is closely associated with atrial fibrillation, thromboembolic risk, and adverse clinical outcomes, thereby contributing to risk stratification and timing of intervention [65]. Morphological features including leaflet thickening, commissural fusion, and subvalvular involvement are characteristic and guide therapeutic decision-making, particularly suitability for percutaneous mitral commissurotomy. In contrast, degenerative MS is characterized by extensive mitral annular calcification, often limiting echocardiographic assessment due to acoustic shadowing. In this setting, planimetric assessment is less reliable, and transesophageal echocardiography and cardiac computed tomography play an important complementary role, particularly for anatomical characterization and procedural planning [61]. Degenerative MS frequently coexists with MR, and observational data indicate that concomitant MR is independently associated with worse survival [66].

#### Implication on timing of valve intervention

Management focuses on relieving obstruction and preventing irreversible pulmonary vascular and RV remodeling. Medical therapy is supportive and includes diuretics for congestion, rate control and anticoagulation for atrial fibrillation. Timely intervention is particularly important before irreversible pulmonary vascular remodeling and advanced RV dysfunction develop, as both substantially limit the potential for reverse remodeling and are associated with adverse clinical outcomes. In symptomatic severe rheumatic MS with favorable valve anatomy, percutaneous balloon mitral commissurotomy is the preferred intervention when MR is mild or less; it may also be considered in selected asymptomatic patients with severe MS and pulmonary hypertension or high embolic risk. Mitral valve replacement is indicated when commissurotomy is unsuitable or unsuccessful, particularly with extensive calcification, significant MR, unfavorable valve morphology, or concomitant indication for cardiac surgery [1, 60, 67]. In high-risk patients with failed bio-prostheses, prior dysfunctional rings, or severe mitral annular calcification, valve-in-valve, valve-in-ring, and valve-in-mitral annular calcification transcatheter approaches are emerging alternatives, although evidence remains limited and anatomy-driven.

## Tricuspid valve disease

### Tricuspid regurgitation

#### Pathophysiology and myocardial remodeling

Tricuspid regurgitation (TR) imposes chronic volume overload on the right heart and gives rise to a progressive right-sided myocardial and venous remodeling. Primary TR results from intrinsic leaflet or subvalvular pathology, including congenital abnormalities, infective endocarditis, rheumatic disease or trauma. In contrast, most clinically relevant TR is secondary and results from right-sided chamber remodeling, annular dilatation, or device-lead interaction [68–70]. Similar to MR, TR is therefore not a uniform disease entity, but rather a heterogeneous syndrome in which the underlying myocardial substrate strongly influences clinical presentation and prognosis [68–70]. Secondary TR has become considerably more refined in recent years. Ventricular secondary TR is often caused by elevated pulmonary pressures and characterized by RV dilatation , whereas atrial secondary TR is driven predominantly by right atrial and annular dilatation in the setting of atrial fibrillation or HF with preserved ejection fraction [68, 70, 71]. This distinction reflects pathophysiologically different remodeling phenotypes with distinct hemodynamic profiles, and clinical outcomes [70–72].

In the compensated phase, the RV may accommodate substantial regurgitant volume while preserving systolic performance. Over time, chronic volume overload promotes a transition from adaptive remodeling to maladaptive right-sided cardiomyopathy characterized by RV dilatation, reduced contractile reserve, and progressive RV-pulmonary artery uncoupling [73, 74]. As disease advances, venous congestion becomes increasingly relevant and is accompanied by worsening exercise intolerance, renal dysfunction, hepatic injury, and systemic fluid retention [73]. These observations support the concept that advanced TR should not be viewed as an isolated valvular lesion, but rather as a broader syndrome of right-sided myocardial, venous, and end-organ remodeling [68, 73, 74]. Beyond the ventricle, the atrium has emerged as an important disease component. Contemporary data indicate that right atrial enlargement and impaired right atrial reservoir function are associated with worse outcome in patients with secondary TR, even after adjustment for other markers of disease severity [75].

#### Multimodality imaging to assess myocardial damage

Multimodality imaging plays a central role in the evaluation of TR, not only for defining lesion severity and mechanism but also for characterizing the extent of myocardial damage and right-sided remodeling. TTE remains the first-line imaging modality and provides information on valve anatomy, regurgitation severity, RV and right atrial size, tricuspid annular dimensions, pulmonary pressures, and vena cava congestion [47]. However, as in other regurgitant lesions, conventional ejection-phase indices may remain preserved until relatively advanced disease, thereby limiting their sensitivity for early myocardial dysfunction. Among echocardiographic parameters, RV free-wall longitudinal strain has emerged as one of the most sensitive markers of myocardial dysfunction in significant TR. In contrast to tricuspid annular plane systolic excursion, fractional area change, or tissue Doppler S′, strain imaging better reflects early impairment of RV systolic mechanics and has shown independent prognostic value beyond conventional indices [76]. In addition, three-dimensional echocardiography may improve quantification of RV volumes and annular geometry, while newer concepts such as regurgitant-volume adjusted or “effective” RV ejection fraction aim to better capture true forward RV performance in the setting of severe regurgitation [77].

CMR has become increasingly important in the assessment of TR-related cardiomyopathy. CMR is the reference standard for RV volumes and systolic function and provides highly reproducible quantification of chamber remodeling and regurgitant burden [47, 78]. Importantly, CMR studies have shown that regurgitant volume and regurgitant fraction carry independent prognostic value in functional TR, highlighting the relevance of quantitative imaging beyond visual grading alone [78]. In addition, CMR enables tissue characterization, and recent data suggest that myocardial fibrosis affecting the interventricular septum identified by LGE may provide incremental information on adverse remodeling and prognosis in advanced functional TR [79]. Emerging imaging approaches have also extended myocardial assessment to systemic consequences of chronic venous congestion, including hepatic tissue remodeling, further emphasizing that advanced TR is a multisystem disease rather than a purely valvular abnormality [80, 81].

Taken together, these observations indicate that imaging in TR should move beyond a narrow focus on regurgitation grade. Instead, comprehensive assessment should include RV systolic function, RV-pulmonary artery interaction, atrial remodeling, chamber dilatation, venous congestion, and tissue-level markers of myocardial or extracardiac injury [47, 78].

#### Implication on timing of valve intervention

Historically, isolated tricuspid valve surgery has been associated with substantial perioperative morbidity and mortality, largely reflecting the fact that patients were referred late, in the presence of advanced right-heart failure, systemic congestion, and end-organ dysfunction [82]. This experience has fundamentally shaped contemporary thinking. The challenge in TR is not only to identify patients who may benefit from intervention, but also to recognize them before remodeling has become irreversible [73, 82].

Current guideline and consensus documents increasingly reflect this stage-based view of disease. The 2025 ESC/EACTS VHD guidelines emphasize early referral, structured assessment of RV function and pulmonary pressures, and careful identification of patients before severe RV dysfunction or precapillary pulmonary hypertension become prohibitive [1]. Similarly, the 2025 ACC Expert Consensus Decision Pathway proposes a dedicated framework for chronic secondary TR centered on systematic identification, phenotyping, risk assessment, treatment selection, and follow-up [83]. Together, these documents mark a shift away from a purely lesion-based approach toward a broader model that incorporates the myocardial and systemic consequences of TR [1, 83].

This conceptual shift is supported by contemporary interventional studies. In the randomized TRILUMINATE trial, tricuspid transcatheter edge-to-edge repair (T-TEER) reduced TR severity and improved symptoms and quality of life compared with medical therapy, with longer-term data suggesting a reduction in heart-failure hospitalization over follow-up [84, 85]. The TRI.Fr trial provided further randomized evidence that T-TEER plus optimized medical therapy improves clinical status in patients with severe symptomatic isolated TR [84]. In parallel, TRISCEND II showed that transcatheter tricuspid valve replacement was superior to medical therapy alone for a hierarchical component endpoint, driven largely by more complete elimination of TR and greater improvement in symptoms and health status, albeit at the cost of relevant procedure-related trade-offs such as pacemaker implantation [85]. Real-world data from the bRIGHT study further support the effectiveness and safety of contemporary T-TEER in patients with advanced disease [86].

Nevertheless, not all patients derive equal benefit from intervention. Accumulating evidence suggests that advanced right-heart failure, severe RV dysfunction, marked pulmonary vascular disease, RV-pulmonary artery injury and clinically relevant hepatic or renal injury identify patients in whom the potential for reverse remodeling may already be limited [1, 74, 76, 82]. Conversely, patients with severe symptomatic TR, preserved or only moderately impaired RV function, limited pulmonary vascular disease, and the absence of advanced end-organ dysfunction appear most likely to derive benefit from transcatheter intervention. Thus, the therapeutic window in TR likely lies before overt decompensation, at a stage when the valve remains anatomically treatable and the myocardium retains sufficient reserve for recovery [1, 82, 83]. In this regard, multimodality imaging, including assessment of RV function, RV-pulmonary artery coupling, right atrial remodeling, and markers of systemic congestion, may become increasingly important to identify the optimal timing of intervention. While timely intervention aims to prevent irreversible right-sided remodeling, medical therapy remains the cornerstone of the management of TR-related cardiomyopathy and right-sided HF, as TR regression may be achieved under appropriate HF pharmacotherapy [87]. Therefore, guideline-directed HF therapy should be implemented according to the underlying HF phenotype, while diuretics represent the mainstay of symptom relief by reducing systemic congestion. In patients with atrial secondary TR, rhythm control strategies and optimal management of atrial fibrillation may promote favorable reverse remodeling and reduce TR severity [88, 89]. Accordingly, current guidelines recommend a period of optimized medical therapy before referral for tricuspid valve intervention whenever clinically appropriate [1]. However, dedicated randomized studies investigating disease-modifying medical therapy specifically in TR are currently lacking.

## Multiple and mixed valvular heart disease (MMVD)

MMVD represents the most complex form of valvular cardiomyopathy, in which interacting valve lesions and heterogeneous loading conditions accelerate myocardial remodeling. Common causes include rheumatic disease, degenerative calcific disease, prior endocarditis and congenital abnormalities. Clinical scenarios include severe AS accompanied by secondary MR and TR due to chronic pressure overload and pulmonary hypertension, combined mitral and tricuspid valve disease, or other mixed left- and right-sided valve lesions, each characterized by distinct remodeling patterns and therapeutic challenges. These combinations frequently accelerate cardiac remodeling through interacting pressure and volume overload, and substantially complicate clinical assessment and therapeutic decision-making [90, 91]. One lesion may mask or exaggerate another lesion, such as severe MR lowering forward stroke volume leading to underestimation of AS gradients [92]. Consequently, identifying the dominant lesion represents one of the major challenges in MMVD, as treatment of the primary hemodynamic lesion may substantially alter the severity and clinical relevance of concomitant valve disease [90].

Assessment requires integration of clinical findings, multimodality imaging, and, whenever necessary, invasive hemodynamics [93]. TTE is first-line, but transesophageal echocardiography, CMR, computed tomography, and invasive hemodynamics are often needed to define lesion severity, chamber remodeling, fibrosis, and procedural planning. In particular, CMR enables accurate quantification of ventricular volumes, regurgitant volumes, and myocardial fibrosis, facilitating assessment of the individual contribution of each valve lesion to the overall hemodynamic burden [48].

Management decisions should be performed individually by a multidisciplinary Heart Team. Medical therapy is primarily directed toward relief of congestion, treatment of arrhythmias, optimization of HF therapy, and management of comorbidities [1, 2]. However, in MMVD mechanical correction of the dominant lesion is usually required before irreversible multi-chamber myocardial damage develops to ensure durable clinical benefit. Surgery remains the reference strategy in MMVD as multiple lesions can be corrected safely and durably in one procedure. Staged or combined transcatheter approaches are increasingly used to provide individualized treatment strategies for selected high-risk patients [94].

## Cardiac amyloidosis and valvular heart disease

The interaction between cardiac amyloidosis (CA) and VHD is increasingly recognized as clinically relevant rather than incidental [95, 96]. Amyloid deposition affects not only the myocardium but may also involve valvular and subvalvular structures, while the restrictive hemodynamic phenotype of CA promotes annular dilatation, chamber remodeling, and progressive valvular insufficiency. Accordingly, VHD in CA is best understood as part of a broader infiltrative cardiomyopathic process rather than an isolated structural valve disorder [95, 96].

Among all valvular lesions, the overlap between AS and transthyretin-associated cardiomyopathy (ATTR-CM) is the most extensively studied [97–102]. Contemporary screening studies in elderly patients referred for TAVR have shown that concomitant ATTR-CM is not rare, affecting ~ 10% of patients [100]. Patients with dual AS-ATTR pathology typically present with greater wall thickness, more frequent low-flow low-gradient hemodynamics, and more advanced myocardial remodeling [95, 96, 100]. Importantly, AVR should not be withheld solely because ATTR-CM is present, as TAVR remains feasible and improves prognosis, as does the use of ATTR-specific medication [103].

Beyond AS, valvular dysfunction appears common across the spectrum of CA [95, 96]. In a large contemporary cohort of 538 patients with CA, approximately 55% presented with at least moderate-to-severe MR and/or TR: isolated MR in 20.8%, isolated TR in 12.3%, and combined MR and TR in 22.3%, with TR carrying the highest adjusted risk among the regurgitant lesions. Other recent cohort data similarly reported a high prevalence of valvular abnormalities in CA and confirmed that more advanced MR or TR identifies patients with worse survival [104].

In the largest serial echocardiographic cohort to date (*n* = 877 patients with ATTR-CM), ≥moderate TR was present in 26.2% at baseline (27.9% in ATTR vs. 23.4% in AL), and patients with moderate-to-severe TR had significantly reduced median survival compared with those with no or mild TR (2.3 vs. 3.35 years, *p* = 0.015).^104^ Crucially, among all echocardiographic parameters tracked longitudinally, progressive worsening of MR and TR emerged as the only valvular changes consistently associated with mortality [104]. Amyloid deposition within the valvular apparatus itself, including leaflet thickening, a shortened and restricted posterior mitral valve leaflet, and thickened chordae tendineae, has also been shown to be a characteristic structural signature of ATTR-CM and contributes to the development of regurgitation independent of annular or chamber dilatation [96].

Importantly, the true prevalence of significant MR in ATTR-CM may be systematically underestimated when conventional (semi-)quantitative echocardiographic criteria are applied, given the restrictive low-flow hemodynamics inherent to the disease. In a recent multicenter cohort of 1,101 patients with newly diagnosed ATTR-CM undergoing standardized core-laboratory echocardiographic assessment, significant (≥ moderate) concomitant TR was present in 36.1% of patients at diagnosis, while only 12–13% met conventional AHA/ACC, ESC/EACTS, or ASE criteria for severe MR, whereas a validated disease-specific, risk-based framework reclassified 21% as severe [105]. Spline-derived prognostic thresholds were substantially lower than current guideline cut-offs (high-risk: EROA > 0.25 cm², regurgitant volume > 38 mL, regurgitant fraction > 35%), reflecting the fact that even modest regurgitant volumes may represent a substantial portion of the effective stroke volume in a low-flow state. The proposed framework outperformed all guideline-based definitions for prediction of both mortality and HF hospitalization, supporting the need for disease-specific MR grading in ATTR-CM.^105^ Taken together, these findings suggest that valvular regurgitation in CA is not merely an innocent bystander of restrictive cardiomyopathy, but rather a marker, and likely mediator, of disease progression [104–107].

Interventional data outside the AS field remain limited but are beginning to emerge. Our group and others have previously shown that M-TEER is safe and effective in reducing MR in patients with ATTR-CM [108, 109]. Notably, systematic screening of patients referred for M-TEER has revealed a prevalence of clinically manifest CA of 11.7%, with an additional 7.5% showing early amyloid infiltration, highlighting the substantial clinical overlap between structural valve disease and infiltrative cardiomyopathy.^108^ Whether M-TEER plus optimized medical therapy yields clinical benefit compared to medical therapy alone in ATTR-CM is currently being investigated in the randomized controlled MILLENNIAL trial (NCT06075823). For TR, initial experience with T-TEER in ATTR-CM likewise suggests procedural feasibility and symptomatic improvement, but current evidence is limited to case reports and small case series [110, 111]. More broadly, these evolving interventional data should be interpreted in the context of an enriched treatment armamentarium that may complement disease-modifying treatment for ATTR-CM, which has demonstrated sustained cardiovascular benefits in phase 3 trials [112].

Collectively, the available evidence supports consideration of CA which should lead to prompt screening in elderly patients with VHD and the presence of red flag signs indicative of CA and favors an integrated management strategy that addresses both the valvular lesion and the underlying infiltrative cardiomyopathy.

## Data Availability

No datasets were generated or analysed during the current study.

## Abbreviations

AR: Aortic regurgitation
AS: Aortic stenosis
ATTR–CM: Transthyretin–associated cardiomyopathy
AVR: Aortic valve replacement
CA: Cardiac amyloidosis
CMR: Cardiac magnetic resonance imaging
GLS: Global longitudinal strain
HF: Heart failure
LGE: Late gadolinium enhancement
LV: Left ventricle
LVEF: Left ventricular ejection fraction
M-TEER: Mitral transcatheter edge-to-edge repair
MMVD: Multiple and mixed valvular heart disease
MR: Mitral regurgitation
MS: Mitral stenosis
RV: Right ventricle
T-TEER: Tricuspid transcatheteredge-to-edge repair
TAVR: Transcatheter aortic valve replacement
TR: Tricuspid regurgitation
SAVR: Surgical aortic valve replacement
TTE: Transthoracic echocardiography
VHD: Valvular heart disease
